# Thyroid-stimulating hormone levels in euthyroid patients 8 years following bariatric surgery

**DOI:** 10.1038/s41366-021-01058-z

**Published:** 2022-01-07

**Authors:** Anne Lautenbach, Marie Wernecke, Oliver Mann, Philipp Busch, Tobias B. Huber, Fabian Stoll, Jens Aberle

**Affiliations:** 1 III Department of Medicine, University Medical Center Hamburg-Eppendorf, Hamburg, Germany; 2 Department of General, Visceral and Thoracic Surgery, University Medical Center Hamburg-Eppendorf, Hamburg, Germany

**Keywords:** Obesity, Endocrine system

## Abstract

**Background:**

Bariatric surgery (BS) was shown to promote a decline in thyroid-stimulating hormone (TSH) in euthyroid patients with severe obesity in the short-term. Aim of the present study was to assess the effect of weight loss on thyroid function in euthyroid patients in the long-term following different bariatric procedures.

**Methods:**

In a retrospective cohort study including 135 patients at baseline, thyroid function was assessed at six time points up to 8 years after surgery. Patients were stratified by TSH levels at baseline and divided into two groups to compare the change in TSH at long-time. We used log-linear regression to assess the relation between thyroid hormones and TSH and linear regression analyses to identify variables that were thought to determine TSH and fT3/fT4-ratio as well as their change long-term.

**Results:**

Over a mean follow-up of 8 years, TSH and fT3/fT4-ratio declined (both *p* < 0.001). Patients with high-normal TSH showed a greater decline in TSH than those with normal TSH compared to baseline. Thyroid hormones and TSH displayed a negative log-linear correlation at long-term follow-up. Change in TSH at long-time showed a negative correlation with TSH at baseline (*B* = −0.55; *p* < 0.001). With regard to type of surgery, there were no significant differences in TSH.

**Conclusion:**

BS promotes a decline of TSH in euthyroid patients up to 8 years after intervention despite weight regain. The greatest change in TSH was seen among patients with high-normal baseline-TSH. Results of log-linear regression suggest recovery of the pituitary-thyroid axis. Type of surgery did not affect the change in TSH levels over time.

## Introduction

In the last few decades the prevalence of obesity constantly increased reaching pandemic levels. The WHO reported a prevalence of 13% for adults with obesity in 2016 [1]. Besides commonly used lifestyle interventions bariatric surgery (BS) has become an effective treatment option for patients with severe obesity [2].

Severe obesity has previously shown to be associated with high-normal serum levels of thyroid stimulating hormone (TSH) and free triiodothyronine (fT3) in euthyroid adults [3]. Subtle changes in thyroid hormones, even within the normal range, adversely influence cardiovascular, bone and metabolic outcomes [4, 5]. High-normal TSH-values are associated with an adverse lipid profile, high blood pressure, high body mass index (BMI), metabolic syndrome and fatal coronary heart disease [4]. However, the causal relationship between severe obesity and thyroid function still remains unclear [6, 7]. Patients with severe obesity may have transient hypothyroidism caused by an increase in leptin-levels that resolves following Roux-en-Y gastric bypass (RYGB) [8].

There are only few studies addressing the relation between change in TSH levels and change in body weight in the euthyroid range following BS [9–14], even though the majority of bariatric patients have normal thyroid function [8]. Short-term studies have shown that weight loss induced by BS promotes a significant decline of increased TSH levels in euthyroid patients with severe obesity [9–11]. This initial decline was progressive over time and associated with excess BMI loss [9]. However, in most studies, thyroid parameters were analyzed after up to 12 months post-surgery [9–13, 15]. Only three studies had a follow-up of 24 months [14, 16, 17]. To the best of our knowledge, there are no studies addressing variations in TSH within the euthyroid range following BS in the long-term. Moreover, no data exist investigating the impact of surgery technique on thyroid metabolism in the long-term.

Therefore, the primary aim of the study was to investigate the effect of weight loss on TSH levels in euthyroid patients in the long-term following different bariatric procedures.

## Materials and methods

### Study population

Euthyroid male or female patients ≥18 years who underwent either sleeve gastrectomy (SG) or RYGB according to the S3 Guidelines *Chirurgie der Adipositas* [18] were included in the analysis. Patients with second-step procedures were considered as having SG (*n* = 2). Patients attended our obesity outpatient clinic between 2008–2020, which is certified as center of excellence for obesity and metabolic surgery by the European Accreditation Council for Bariatric Surgery. Exclusion criteria included incomplete records, history of autoimmune thyroid disease, history of acute inflammation (pulmonary, gastrointestinal, urogenital, or cutaneous), history of chronic autoinflammatory disease, pituitary insufficiency, pregnancy and treatment with thyreostatic drugs, thyroid hormones, amiodarone or lithium. Blood samples were taken in the afternoon in a non-fasting condition. Of an initial population of 330 patients, 195 were excluded (*n* = 135).

All procedures performed in this study involving human participants were in accordance with the ethical standards of the institutional and/or national committee on human research and with the 1964 World Medical Association Declaration of Helsinki and its later amendments or comparable ethical standards. Retrospective data collection and anonymized analysis was conducted in accordance with local government law (HmbKHG. §12) without the requirement for informed consent.

### Study design

Follow-up data were retrospectively collected from 135 patients at baseline. To provide reasonable comparability between the cases, the available data were allocated 5 “visits” by time in relation to the procedure. In addition to baseline data 8.07 ± 5.26 (mean ± SD) months before surgery (*n* = 135), data from visit 1 (*n* = 132) were analyzed 6 months after surgery, data from visit 2 (*n* = 110) 1 year after surgery, data from visit 3 (*n* = 97) 2 years after surgery, data from visit 4 (*n* = 72) 3 years after surgery and data from visit 5 (*n* = 52) 8 years post BS. Over the follow-up period 82 patients were lost to follow-up.

### Variables

Data on height, weight, BMI, sex, age, and serum levels of TSH, fT3, free thyroxine (fT4), glycated hemoglobin (HbA1c), total cholesterol, triglycerides, low-density lipoprotein cholesterol (LDL-cholesterol), high-density lipoprotein cholesterol (HDL-cholesterol), C-reactive protein (CRP), aspartate aminotransferase (AST), alanine aminotransferase (ALT), and gamma-glutamyl transferase (GGT) were analyzed at baseline and during follow-up. Data on cardiovascular comorbidities were recorded at baseline and data on post-surgical complications were recorded over the follow-up period. fT3 and fT4 were measured by a sandwich chemiluminescence homogeneous immunoassay (LOCI-technology, Dimension Vista 1500 Analyzer, Siemens Healthineers). The normal range for serum fT3 levels and fT4 levels was 3.5–6.5 pmol/l and 11.5–22.7 pmol/l, respectively. TSH was measured by a sequenced chemiluminescence homogeneous immunoassay (LOCI-technology, Dimension Vista 1500 Analyzer, Siemens Healthineers). The normal range for serum TSH levels was 0.55–4.78 mU/l. The fT3/fT4-ratio as an indirect index of D1 and D2 deiodinase activity was calculated [16]. Excess weight loss (EWL) in % was calculated by dividing the difference between initial and final BMI by the difference between initial BMI and a target BMI of 25 kg/m². Optimal weight loss was defined as losing at least 50% of the excess weight during the first 2 years after the procedure [19, 20]. We computed the change in TSH levels and fT3/fT4-ratio as the change between baseline and each follow-up visit.

### Subgroup classification

Patients were stratified by TSH-levels at baseline and divided into two groups based on previous studies that indicate that more than 95% of healthy individuals have TSH levels below 2.5 mU/l [21, 22]: (1) normal TSH (<2.5 mU/l) and (2) high-normal TSH (≥2.5 mU/l). We also performed subgroup analyses to compare the change in thyroid function between different surgical approaches (SG vs. RYGB) as well as the presence and absence of treatment requiring hypertension.

### Statistical analysis

Independent continuous variables were analyzed fitting a mixed-effects model for repeated measures treating missing values of lost to follow-up patients as random effect. The model uses a compound symmetry covariance matrix and is fit using restricted maximum likelihood. Tukey’s test was used to correct for multiple comparisons. Non-sphericity of variables was corrected using the Geisser–Greenhouse method. Subgroups were added as additional factor to the model using Fisher’s least significant difference test for comparisons between subgroups.

Log-linear regression between log-transformed TSH-levels and thyroid hormones was performed for each visit. Linear regression and multiple linear regression analysis were performed to identify independent variables that determined TSH-levels and fT3/fT4-ratios as well as their changes compared to baseline for all visits and compared to visit 4 at long-term.

Continuous variables are expressed as mean ± SD. Categorical variables are presented as absolute or relative numbers. For all statistical tests a *p* < 0.05 (*p < 0.05, **p < 0.01 and ***p < 0.001) was considered statistically significant. A minimal sample size of 53 was calculated assuming an effect size of Cohen’s *f* = 0.25 with a type I error of *α* = 0.05, a statistical power of 0.95, number of repeated measurements of 6, a correlation among repeated measures of 0.5 and an assumed correction for non-sphericity of *ε* = 0.4 with G*Power, version 3.1.9.7 for Windows [23]. All analysis and graphical representation were performed via GraphPad Prism 9, version 9.2.0 for Windows, GraphPad Software Inc., California, United States.

## Results

### Demographic and biochemical data follow-up at baseline

Mean age at baseline was 42.1 ± 10.6 years, 68.9% of patients were female. In total, 45.2% of patients underwent SG, 54.8% RYGB as initial weight loss procedure. Demographic data for baseline and follow-up visits is presented in Table 1. At baseline, 49.8% of patients suffered from treatment requiring hypertension, 2.2% from coronary heart disease, and 3.0% from cardiac arrythmia.

**Table 1:** Demographic characteristics of study population at baseline and follow-up visits.

	Baseline	Visit 1	Visit 2	Visit 3	Visit 4	Visit 5
*N*	135	132	110	97	72	52
Sex (F, *n*)	93	92	76	71	55	39
Age (years)	42.1 ± 10.6	43.3 ± 10.4	43.8 ± 10.5	45.6 ± 9.3	46.1 ± 9.8	52.6 ± 8.9
SG (*n*)	61	58	51	44	33	22
RYGB (*n*)	74	74	59	53	39	30
Time to BS (months)	−8.07 ± 5.26	6.06 ± 1.19	11.90 ± 1.32	23.32 ± 2.60	35.80 ± 2.57	95.60 ± 6.97
Optimal weight loss (%)	71.9	72.0	80.3	72.2	75.0	76.9

Data are reported as mean ± SD.

*N* number of individuals, *F* female, *SG* sleeve gastrectomy, *RYGB* Roux-en-Y gastric bypass, *BS* bariatric surgery.

Post-surgical complications included the development of dumping syndrome (5.9% of patients) as well as gastroesophageal reflux disease (1.5% of patients).

At baseline, mean BMI was 50.8 ± 8.6 kg/m² and HbA1c was 6.3 ± 1.2%. At baseline, TSH was 2.06 ± 0.79 mU/l, fT3/fT4-ratio was 0.33 ± 0.05. Biochemical data for baseline and follow-up visits is presented in Table 2.

**Table 2:** Anthropometric and biochemical characteristics at baseline and follow-up visits.

	Baseline	Visit 1	Visit 2	Visit 3	Visit 4	Visit 5
Weight (kg)	150.8 ± 34.2	115.3 ± 27.7***	109.6 ± 30.0***	105.4 ± 26.1***	103.4 ± 26.4***	114.6 ± 30.8***
BMI (kg/m²)	50.8 ± 8.6	38.9 ± 7.3***	36.9 ± 8.2***	35.8 ± 7.5***	35.3 ± 7.8***	38.6 ± 8.5***
EWL (%)		48.9 ± 16.4	58.7 ± 22.4	60.3 ± 23.6	63.1 ± 23.0	46.1 ± 34.7
TSH (mU/l)	2.06 ± 0.79	1.61 ± 0.78***	1.55 ± 0.70***	1.32 ± 0.62***	1.34 ± 0.56***	1.35 ± 0.62***
fT3 (pmol/l)	5.0 ± 0.6	4.5 ± 0.6***	4.4 ± 0.6***	4.4 ± 0.6***	4.3 ± 0.5***	4.0 ± 0.6***
fT4 (pmol/l)	15.4 ± 2.4	16.2 ± 2.7**	16.0 ± 2.1*	16.2 ± 1.9*	16.5 ± 2.1***	15.9 ± 2.3
fT3/fT4	0.33 ± 0.05	0.28 ± 0.05***	0.28 ± 0.04***	0.28 ± 0.04***	0.26 ± 0.04***	0.25 ± 0.04***
HbA1c (%)	6.3 ± 1.2	5.6 ± 0.7***	5.6 ± 0.7***	5.8 ± 1.0***	5.6 ± 1.1***	5.9 ± 1.1**
Total cholesterol (mg/dl)	207 ± 35	189 ± 36***	188 ± 33***	189 ± 33***	186 ± 33***	191 ± 32***
Triglycerides (mg/dl)	203 ± 123	138 ± 82***	140 ± 70***	145 ± 72***	128 ± 70***	141 ± 73***
LDL-cholesterol (mg/dl)	119 ± 30	112 ± 33**	101 ± 27***	100 ± 32***	98 ± 29***	99 ± 30***
HDL-cholesterol (mg/dl)	51 ± 15	51 ± 14	57 ± 16***	61 ± 18***	64 ± 21***	62 ± 16***
CRP (mg/l)	10.2 ± 7.4	7.0 ± 4.6***	6.1 ± 2.8***	5.9 ± 2.8***	6.3 ± 3.7***	5.4 ± 2.2***
AST (U/l)	33 ± 20	23 ± 9***	20 ± 8***	19 ± 7***	20 ± 10***	20 ± 6***
ALT (U/l)	38 ± 25	24 ± 13***	20 ± 10***	20 ± 8***	21 ± 11***	26 ± 9***
GGT (U/l)	44 ± 33	27 ± 20***	25 ± 16***	26 ± 19***	27 ± 22***	24 ± 12***

Data are reported as mean ± SD. A mixed-effects model with Tukey’s multiple comparisons test was used to compare follow-up visits with baseline for continuous variables (*p < 0.05, **p < 0.01 and ***p < 0.001).

*BMI* body mass index, *EWL* excess weight loss, *TSH* thyroid stimulating hormone, *fT3* free triiodothyronine, *fT4* free thyroxine, *HbA1c* glycated hemoglobin, *LDL* low-density lipoprotein, *HDL* high-density lipoprotein, *CRP* C-reactive protein, *AST* aspartate aminotransferase, *ALT* alanine aminotransferase, *GGT* gamma-glutamyl transferase.

Comparing SG and RYGB, TSH was not significantly different between the groups at baseline (2.06 ± 0.84 vs. 2.07 ± 0.75 mU/l, *p* = 0.90).

Comparing between the presence and absence of treatment requiring hypertension at baseline, there was a slight difference by trend in TSH between the groups at baseline, although not statistically significant (1.96 ± 0.69 vs. 2.16 ± 0.87, *p* = 0.098).

When evaluating by baseline-TSH, the groups did not differ significantly in any other parameter than TSH. Normal TSH-levels with a mean of 1.63 ± 0.45 mU/l was present in 68.9% of patients. Patients were 41.9 ± 12.1 years, BMI was 50.7 ± 8.7 kg/m², HbA1c was 6.3 ± 1.2%. High-normal TSH-levels with a mean of 3.02 ± 0.47 mU/l was present in 31.1% of patients. Patients were 42.2 ± 9.87 years, BMI was 51.0 ± 8.4 kg/m², HbA1c was 6.2 ± 1.2%.

### Weight loss outcomes 8 years after surgery

Body weight and BMI declined during follow-up compared to baseline (*p* < 0.001, all visits). Weight regain at visit 5 was statistically significant compared to visit 4 (*p* < 0.001) (Table 2). The mean EWL at long-term was 46.1 ± 34.7%. At visit 5, 76.9% of patients had achieved optimal weight loss of greater than 50% EWL during the first 2 years after surgery (Table 1).

Comparing SG and RYGB, EWL was not significantly different between the groups at long-term (43.3 ± 13.7 vs. 48.1 ± 44.4%, *p* = 0.58).

There was no significant difference between patients with normal and high-normal TSH baseline-levels in body weight (116.4 ± 31.3 vs. 110.9 ± 30.3 kg, *p* = 0.55), BMI (39.3 ± 8.6 vs. 37.2 ± 8.5 kg/m², *p* = 0.41) and EWL (41.2 ± 39.5 vs. 56.2 ± 19.2%, *p* = 0.07) at visit 5.

### Glycemic control 8 years after surgery

HbA1c significantly decreased over the whole follow-up period compared to baseline (*p* < 0.001, visit 1–4; *p* = 0.005, visit 5). A gradual re-increase occurred at long-term, which was not statistically significant compared to visit 4 (*p* = 0.054) (Table 2).

Comparing SG and RYGB, HbA1c was not significantly different between the groups at long-term (5.7 ± 0.8 vs. 6.0 ± 1.2%, *p* = 0.26).

When evaluating by baseline-TSH, HbA1c did not differ significantly between patients with normal and high-normal baseline-levels at visit 5 (5.6 ± 0.8 vs. 6.0 ± 1.1%, *p* = 0.16).

### Metabolic and cardiovascular parameters 8 years after surgery

Total cholesterol and triglycerides decreased over the whole follow-up-period compared to baseline (*p* < 0.001, all visits) (Table 2). A gradual re-increase at long-term was not statistically significant compared to visit 4 for both parameters (*p* = 0.214 and *p* = 0.053). LDL-cholesterol statistically decreased during follow-up compared to baseline (*p* = 0.007, visit 1; *p* < 0.001, visit 2–5), while HDL-cholesterol remained stable at visit 1 compared to baseline (*p* = 0.498) and statistically increased over the remaining follow-up period compared to baseline (*p* < 0.001, visit 2–5) (Table 2).

Mean serum levels of CRP statistically declined over the whole follow-up period compared to baseline (*p* < 0.001, all visits) (Table 2) and showed a statistically significant re-increase at long-term compared to visit 4 (*p* = 0.013).

Serum levels of AST, ALT, and GGT dropped over the whole follow-up period compared to baseline (*p* < 0.001, all visits) (Table 2). ALT showed a statistically significant re-increase at long-term compared to visit 4 (*p* = 0.001), while AST and GGT remained stable at long-term compared to visit 4 (*p* = 0.944 and *p* = 0.107).

### Thyroid function outcomes 8 years after surgery

TSH-levels and fT3/fT4-ratio declined over the follow-up period compared to baseline (*p* < 0.001, all visits). At visit 5, TSH remained stable compared to visit 4 (1.34 ± 0.56 vs. 1.35 ± 0.62 mU/l, *p* = 0.80) and fT3/fT4-ratio remained stable compared to visit 4 (0.26 ± 0.04 vs. 0.25 ± 0.04, *p* = 0.57) (Table 2).

Comparing SG and RYGB, there were no significant differences in TSH (1.25 ± 0.60 vs. 1.43 ± 0.63 mU/l, *p* = 0.29) and fT3/fT4-ratio (0.26 ± 0.04 vs. 0.25 ± 0.03, *p* = 0.33) between the surgical procedures at visit 5.

Comparing between the presence and absence of treatment requiring hypertension at baseline, TSH was not statistically significant different between the groups at visit 5 (1.21 ± 0.59 vs. 1.50 ± 0.59, *p* = 0.152), while fT3/fT4-ratio showed a statistically significant difference at visit 5 (0.24 ± 0.04 vs. 0.27 ± 0.03, *p* = 0.022).

**Fig. 1:**
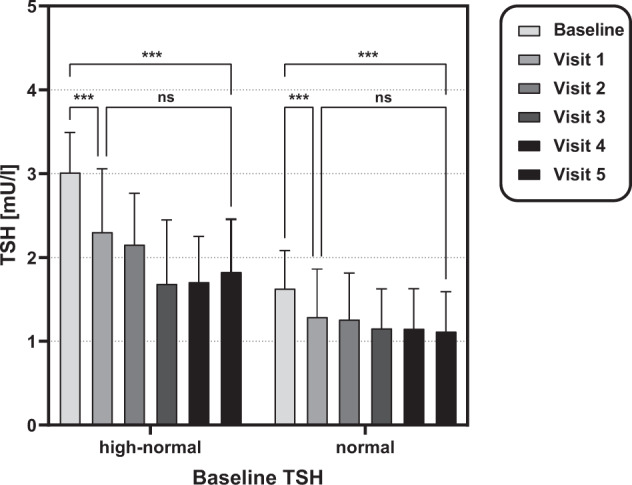
Subgroup classification according to TSH-levels at baseline and postoperative course of TSH for both subgroups. TSH thyroid stimulating hormone. Results are expressed as means and standard deviation. *p < 0.05, **p < 0.01 and ***p < 0.001.

When evaluating by baseline-TSH, TSH-levels as well as the corresponding fT3/fT4-ratios decreased in patients with normal and high-normal TSH baseline-levels over time (*p* < 0.001, all visits). The most pronounced decline in TSH-levels compared to baseline appeared at visit 1 for both groups (1.63 ± 0.45 vs. 1.30 ± 0.57, *p* < 0.001 and 3.02 ± 0.47 vs. 2.31 ± 0.75, *p* < 0.001) and remained stable at long-term follow-up (shown in Fig. 1). Patients with high-normal TSH-levels at baseline showed a greater change in TSH compared to baseline at long-time follow-up than patients with normal TSH-levels at baseline (−1.16 ± 0.70 vs. −0.39 ± 0.51, *p* = 0.002). There was no significant difference in change of fT3/fT4-ratio compared to baseline at visit 5 between the two groups (−0.09 ± 0.04 vs. −0.08 ± 0.05, *p* = 0.54).

### Log-linear regression analysis between TSH and thyroid hormones at long-time follow-up

**Fig. 2:**
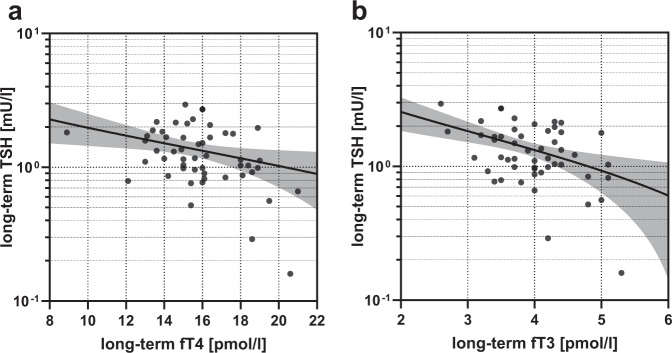
Log-linear regression analysis between TSH and thyroid hormones at long-time follow-up. **a** fT3 and TSH-levels, **b** fT4 and TSH-levels. fT3 free triiodothyronine, fT4 free thyroxine, TSH thyroid stimulating hormone.

Log-linear regression analysis at long-time follow-up showed a negative log-linear correlation between fT3-levels and log-transformed TSH levels (log TSH = 3.78–4.09 fT3, *r*² = 0.19, *p* = 0.001) (shown in Fig. 2a). Likewise, fT4-levels showed a negative correlation with TSH-levels at long-term-follow-up (log TSH = 5.13–3.16 fT4, *r*² = 0.11, *p* = 0.017) (shown in Fig. 2b). Similar log-linear regression analysis at baseline and earlier follow-up visits showed no significant negative log-linear correlation between thyroid hormones and log-transformed TSH-levels, except at 1-year follow-up (log TSH = 3.81–3.52 fT3, *r*² = 0.09, *p* = 0.001 and log TSH = 5.10–2.96 fT4, *r*² = 0.05, *p* = 0.013).

### Linear regression analysis for change in TSH and for change in fT3/fT4-ratio at long-time follow-up

**Fig. 3:**
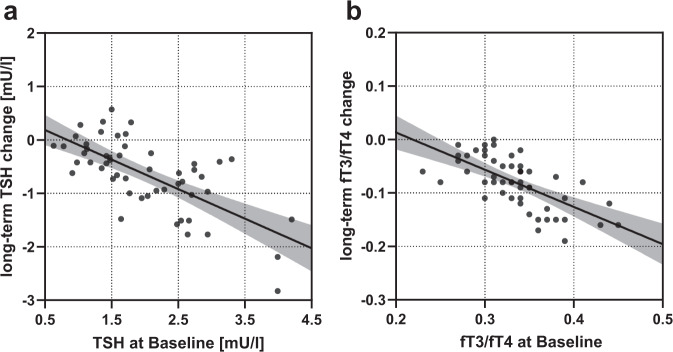
Linear regression analysis for change in TSH and for change in fT3/fT4-ratio at long-time follow-up. **a** Linear regression analysis between TSH-change at long-term follow-up and TSH at baseline and **b** fT3/fT4-change at long-term follow-up and fT3/fT4 at baseline. TSH thyroid stimulating hormone, fT3 free triiodothyronine, fT4 free thyroxine.

Change in TSH and change in fT3/fT4-ratio at long-time follow-up showed a negative correlation with TSH at baseline (shown in Fig. 3a) and fT3/fT4-ratio at baseline (shown in Fig. 3b), respectively (*B* = −0.55, *p* < 0.001 and *B* = −0.70, *p* < 0.001; respectively). There was no statistically significant correlation between weight regain at long-term compared to visit 4 with change in TSH and change in fT3/fT4-ratio at long-time follow-up compared to visit 4 (*B* = 0.005, *p* = 0.622 and *B* = 0.029, *p* = 0.270).

### Multiple linear regression analysis for change in TSH and for change in fT3/fT4-ratio at long-time follow-up

Multiple linear regression showed that TSH at baseline was the only variable significantly associated with the change in TSH at visit 5. Change in TSH at long-time showed a negative correlation with TSH at baseline (*B* = −0.47, *p* < 0.001). Accordingly, fT3/fT4-ratio at baseline was the only variable associated with the change in fT3/fT4-ratio at visit 5. Change in fT3/fT4-ratio at long-time follow-up showed a negative correlation with fT3/fT4-ratio at baseline (*B* = −0.61, *p* < 0.001).

## Discussion

This retrospective study is the first study providing long-term data about thyroid function in euthyroid patients with obesity after BS. We report a significant decline in TSH-levels and fT3/fTt4-ratio 8 years after surgery. Our data add knowledge about velocity, temporal dynamics and interrelationship to available short-term studies, in which a decline in TSH-levels 6–24 months after weight loss surgery has been observed [10, 11, 24, 25].

In the present cohort study, TSH-levels declined by 0.71 mU/l over a mean follow-up of 8 years. The most pronounced decline in TSH was observed during the first 6 months following BS. Observational studies showed that there is a positive association between BMI and TSH, even within the normal range [26, 27]. Causality was assessed by a recent Mendelian randomization analysis, showing that thyroid function correlated to weight increases over 5 years. Thyroid function accounted for 1% of variation in BMI between individuals, having the same impact on BMI as physical activity [28]. In a cross-sectional population study, a difference in BMI of 1.9 kg/m² (5.5 kg) between patients with the highest and lowest TSH-levels, but no overt thyroid dysfunction, was observed [26].

Moreover, change in TSH at long-time showed a strong negative correlation with TSH at baseline, which is in line with data 6–12 months after SG in euthyroid patients [10]. Change in TSH was greater in patients with high-normal TSH values at baseline, suggesting that patients with greater baseline dysregulation will have greater decrease of TSH [13]. Since there is evidence for the association of high-normal TSH with negative cardiovascular and metabolic effects, “normalization” of TSH-levels promoted by BS is particularly important in this at-risk population [29, 30].

In our cohort, serum TSH-levels and thyroid hormones fT3 and fT4 presented a strong negative log-linear relation only at 1-year and long-term follow-up. Negative log-linear correlation between serum TSH-levels and thyroid hormone levels has been well described previously in healthy individuals [22, 31, 32] and reflect the important role of triiodothyronine and thyroxine in the pituitary feedback [33]. Therefore, our data indicates normalization of pituitary feedback and recovery of the pituitary-thyroid-axis 8 years after weight loss surgery. It remains unclear, whether this normalization at long-term was achieved due to weight loss after BS or behavioral and nutritional adjustments during the follow-up period. The impact of nutritional status on the pituitary-thyroid-axis has already been demonstrated before [34–36].

Proposed mechanisms include hypothalamic effects of leptin, which correlates with the percentage of body fat mass and energy balance [13, 35, 37]. TSH-variability in 800 patients with obesity was mainly explained by leptin independently of BMI, suggesting that TSH might be associated with energy balance rather than obesity [37]. Patients with high-normal TSH displayed higher leptin-levels compared to those with normal TSH-values. Following BS, circulating levels of leptin decrease related to weight loss and food restriction leading to reduced stimulation of TRH- and TSH-secretion referred to as the “hypothalamic-pituitary-adipose-axis” by some authors [37, 38]. Suppression of serum ghrelin following RYGB and SG may have an additive effect on the reduction in TSH-levels [10]. Surprisingly, the course of TSH and fT3/fT4-ratio did not follow the U-shaped curve of weight loss. Furthermore, weight regain at long-term showed no association with the changes in TSH-levels, suggesting thyroid function not to mirror the course of weight loss in the long-term. Thyroid axis function has been previously discussed to interact with food intake and energy expenditure on a more central level and therefore regulating energy balance affected by both, peripheral and central tissue [39]. We can only speculate that food restriction and negative energy balance are key to the favorable long-term effect of BS despite gradual weight regain. Long-term studies that could further elucidate the underlying mechanisms are needed for a thorough understanding of these complex interactions.

Comparing SG and RYGB, there were no significant differences between TSH-levels at long-time in our cohort. This may be explained by the fact that EWL did not significantly differ in patients undergoing RYGB compared to SG 8 years post BS. Moreover, TSH at baseline, which must be considered as the main driver for change in TSH-levels, was not significantly different.

## Conclusion

In conclusion, results of the present study reveal a decline in TSH-levels in euthyroid patients with obesity up to 8 years after BS irrespective of type of surgery and despite gradual weight regain.

Our study has a few limitations. First, our study is a retrospective cohort study, so that internal and external validity may be impaired. Repeat measurements of TSH-levels at baseline were not performed. T3 and thyroid antibodies were not included in the assessment. Vitamin and mineral supplementation, glucocorticoid and psychotropic medication, that may have an influence on thyroid function, were not recorded. Analysis of A1C did not include measurement of hemoglobin.
